# Differences between mechanically stable and unstable chronic ankle instability subgroups when examined by arthrometer and FAAM-G

**DOI:** 10.1186/s13018-015-0171-2

**Published:** 2015-03-08

**Authors:** Heinz Lohrer, Tanja Nauck, Dominic Gehring, Sabrina Wissler, Bela Braag, Albert Gollhofer

**Affiliations:** Institute for Sports Medicine, Otto-Fleck-Schneise 10, D-60528 Frankfurt am Main, Germany; Department of Sport and Sport Science, University of Freiburg, Schwarzwaldstraße 175, D-79117 Freiburg, Germany; Johann Wolfgang Goethe-University, Theodor-Stern-Kai 7, D-60590 Frankfurt, Germany

**Keywords:** Chronic ankle instability, Ankle arthrometer, FAAM, Functional ankle instability, Mechanical ankle instability

## Abstract

**Background:**

The objective measurement of the mechanical component and its role in chronic ankle instability is still a matter of scientific debate. We analyzed known group and diagnostic validity of our ankle arthrometer. Additionally, functional aspects of chronic ankle instability were evaluated in relation to anterior talar drawer.

**Methods:**

By manual stress testing, 41 functionally unstable ankles were divided as mechanically stable (*n* = 15) or mechanically unstable (*n* = 26). Ankle laxity was quantified using an ankle arthrometer. Stiffness values from the load displacement curves were calculated between 40 and 60 N. Known group validity and eta^2^ were established by comparing manual and arthrometer testing results. Diagnostic validity for the ankle arthrometer was determined by a 2 × 2 contingency table. The functional ankle instability severity was quantified by the German version of the Foot and Ankle Ability Measure (FAAM-G). Stiffness (40–60 N) and FAAM-G values were correlated.

**Results:**

Mechanically unstable ankles had lower 40–60 N stiffness values than mechanically stable ankles (*p* = 0.006 and <0.001). Eta for the relation between manual and arthrometer anterior talar drawer testing was 0.628. With 5.1 N/mm as cut-off value, accuracy, sensitivity, and specificity were 85%, 81%, and 93%, respectively.

The correlation between individual 40–60 N arthrometer stiffness values and FAAM-G scores was *r* = 0.286 and 0.316 (*p* = 0.07 and 0.04).

**Conclusions:**

In this investigation, the ankle arthrometer demonstrated a high diagnostic validity for the determination of mechanical ankle instability. A clear interaction between mechanical (ankle arthrometer) and functional (FAAM-G) measures could not be demonstrated.

**Electronic supplementary material:**

The online version of this article (doi:10.1186/s13018-015-0171-2) contains supplementary material, which is available to authorized users.

## Background

**Figure 1:**
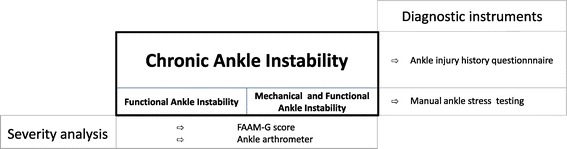
**Schematic for the definitions and the respective test instruments used in the present study.**

The term “chronic ankle instability” (CAI) was introduced in 2002 and is increasingly referenced since then [1]. It is generally agreed that CAI (Figure 1) is an “encompassing term” covering both functional ankle instability (FAI) and mechanical ankle instability (MAI) [1,2]. CAI is “the most commonly used term to describe subjects who report ongoing symptoms after an initial ankle sprain” [3]. Acute lateral ankle sprains have to be differentiated from CAI. It can take from 6 weeks to 3 months for ligament healing to be complete after acute ankle sprain [4]. “Copers” are defined as people who fully recover after an ankle sprain [5].

Within the literature at this time, however, it is obvious that the terms CAI, FAI, and MAI are not precisely defined and thus were not well separated in previous studies. Even recently, these terms have been used synonymously. A “consistent terminology” is demanded [2,3,6]. A systematic investigation has shown that the outcome in research is mainly affected by the definitions of CAI, FAI, and MAI [2]. In consequence, it is therefore proposed to exactly define the inclusion and exclusion criteria when investigating CAI [2,3]. More detailed analyses show that MAI and FAI interaction appears to be essential to get a thorough understanding of the different phenomena of CAI.

The concept of FAI was established in 1966 to describe “the tendency for the foot to give way after an ankle sprain” [7]. To explain “instabilities despite a mechanically stable ankle”, sensorimotor pathway alterations and impaired neuromuscular control are assumed [8], and a broad variety of complaints was included [2]. With respect to the concept of MAI “pathologic laxity, impaired arthrokinematics and synovial and degenerative changes” have also been attributed [9,10].

An Ankle Injury History Questionnaire has been introduced in 2008 to select CAI persons [11]. Recently, the International Ankle Consortium established “selection criteria for patients with chronic ankle instability in controlled research” [3]. Specifically, one significant ankle sprain, followed by “giving way,” and/or recurrent sprain, and/or “feelings of instability” and persisting disability, documented with a self-reported foot and ankle function questionnaire is recommended [3]. The rating of MAI is considered as a “potential confounding factor” [3]. Thus, the impact of MAI remains controversial. Results from our clinical and experimental research [12,13] are in contrast with the assumption that “there has not been a definitive association of ankle laxity with CAI” [3]. Clinical literature nearly exclusively means MAI when using the term “CAI” and operative ligament reconstruction consistently results in both functionally and mechanically stable ankles [12-15].

MAI “is universally accepted as pathologic ligamentous laxity about the ankle-joint complex” [10]. This means that isolated ankle laxity (=hypermobility) without subjectively perceived symptoms is not a pathologic condition and can neither be labeled MAI nor FAI nor CAI [3].

Several instruments have been described to determine the severity of MAI or FAI [6]. There is no generally agreed gold standard to diagnose and quantify MAI [2]. Manual stress testing is widely accepted and proposed as standard to divide mechanically stable from mechanically unstable ankles [6,16-18]. Its accuracy, however, is still debated [19-23]. In the research setting, specific disadvantages of this procedure are rater dependency and its qualitative categorizing nature [10]. Recent literature has proposed to perform studies that “validate manual stress tests with instrumented arthrometry” [24]. Compared with arthroscopy, the sensitivity of anterior talar drawer (ATD) stress radiographs, ultrasound, and MRI to detect chronic anterior talofibular ligament injury was 92%, 100%, and 92%, respectively [25]. However, radiographic stress testing is still a matter of discussion [26,27]. To avoid radiation, several non-radiographic ankle stress testing devices have been developed [6,28]. Published normative values are relevant only for testing performed with one specific apparatus [29]. Generally accepted normative values do not exist, and therefore, the diagnostic use of ankle arthrometers is limited so far. We developed and validated an ankle arthrometer in a cadaveric study and in an *in vivo* pilot study [28,30]. Balance, strength, and self-report function questionnaires have been shown to quantify or to diagnose FAI [9,28].

The main purpose of this study was to perform a “known group validation” [31] for our ankle arthrometer. Additionally, we evaluated the diagnostic validity of our ankle arthrometer. We finally questioned if an interaction exists between the mechanical (ankle arthrometer) and the functional (FAAM-G) measures.

## Methods

This cross-sectional investigation is part of a larger study to experimentally evaluate the impact of CAI on involuntarily foot inversion during gait [13]. The study was approved by the Ethics Commission of the University of Freiburg, Germany and by the Landesärztekammer Hessen Ethics Committee. All participating subjects read and signed the informed consent form.

### Study population

**Figure 2:**
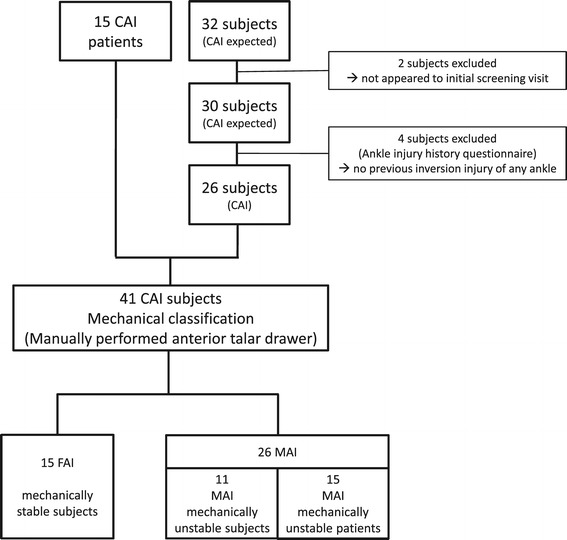
**Flow chart to demonstrate the recruitment procedure of the tested groups under mechanical (MAI) and functional (FAI) considerations.** CAI = chronic ankle instability. MAI = mechanical ankle instability. FAI = functional ankle instability.

By announcement in the local press, 32 males were recruited (Figure 2). We included 26 subjects (Table 1). These “subjects” complained residual symptoms after ankle sprain(s) and were therefore CAI by definition [3]. Compared with these mildly affected CAI subjects, we assumed that patients who were waiting for ankle ligament reconstruction will suffer more severe CAI symptoms [32]. Therefore, 15 consecutive patients were selected in our sports medicine institute to represent a “patients’ group” (Table 1). All these patients were diagnosed with MAI and were already described in a previous paper [32]. All subjects and patients were lower competitive level or recreational athletes (Table 1). Inclusion and exclusion criteria were based on the Ankle Injury History Questionnaire [11]: Subjects and patients were included when they reported at least one of the following criteria: a history of at least one ankle sprain more than 1 year ago. Additionally, actual symptoms of giving way, or feeling of giving way (at least once a month), and/or feelings of instability had to be stated (Table 1). Subjects and patients were separated based on the presence or absence of MAI (Figure 1). Persons with systemic diseases, neuromuscular disorders, and obesity (BMI greater than or equal to 30) were excluded. Subjects who complained of ankle pain as a primary symptom, who had an acute ankle sprain within the past 6 months or had previous foot and ankle surgery, fractures, or anatomic deformities of the lower extremities were also excluded. Persons who presented more than 10 degrees of knee hyperextension in manual testing were also excluded.

**Table 1:** **Anthropometrics and data from the individual histories for the tested group**

**Classification**	**No.**	**Age (years)**	**Height (cm)**	**Weight (kg)**	**Right/left**	**AAS**	**Previous ankle sprains**	**Feeling of instability**	**Pain**	**Limitation**
FAI subjects	15	24.9 ± 2.3	184.7 ± 6.2	84.3 ± 11.2	6/9	8.3 ± 1.2	2.8 ± 3.8	6/9	5/9	6/8
		[22–29]	[174.0–193.0]	[61.6–106.6]		[5–9]	[0–12]			
MAI subjects	11	26.3 ± 4.7	180.1 ± 5.1	77.4 ± 5.5	3/8	7.5 ± 1.3	5.2 ± 5.8	7/7	3/8	3/8
		[20–38]	[173.5–187.0]	[67.5–88.4]		[6–9]	[1–20]			
MAI patients	15	32.9 ± 13.5	175.9 ± 7.6	70.5 ± 14.7	5/10	6.8 ± 2.4	6.2 ± 3.9	11/11	14/14	14/14
		[16–57]	[165.0–190.0]	[58.0–110.0]		[2–9]	[1–10]			

AAS = Ankle activity score [33].

*Pain = *ankle pain during or following physical activities. *Limitation = *restriction to perform physical activities.

### Testing procedure

Included subjects initially filled out the Ankle Injury History [11] and the German version of the Foot and Ankle Ability Measure questionnaire (FAAM-G) to assess the severity of pain and disability. The two subscales of the instrument relate to activities of daily living (standing, walking, squatting, personal hygiene, working, and leisure time activities) and evaluate the ability to play sport. The maximum FAAM-G score is 100 and represents a pain free and unrestricted level of physical function [34]. One FAAM-G questionnaire was filled out per patient. One investigator (TN) was present during this process.

Physical examination was performed and documented by a second independent investigator specialized in foot and ankle (HL). He was blinded to the questionnaires’ results and was unaware about the subjects’ functional ankle status. Mechanical ankle stability was evaluated by manual ATD [16,35]. Each ankle was categorized as “mechanically stable” (=no ATD) or as “mechanically unstable” (=positive ATD).

**Figure 3:**
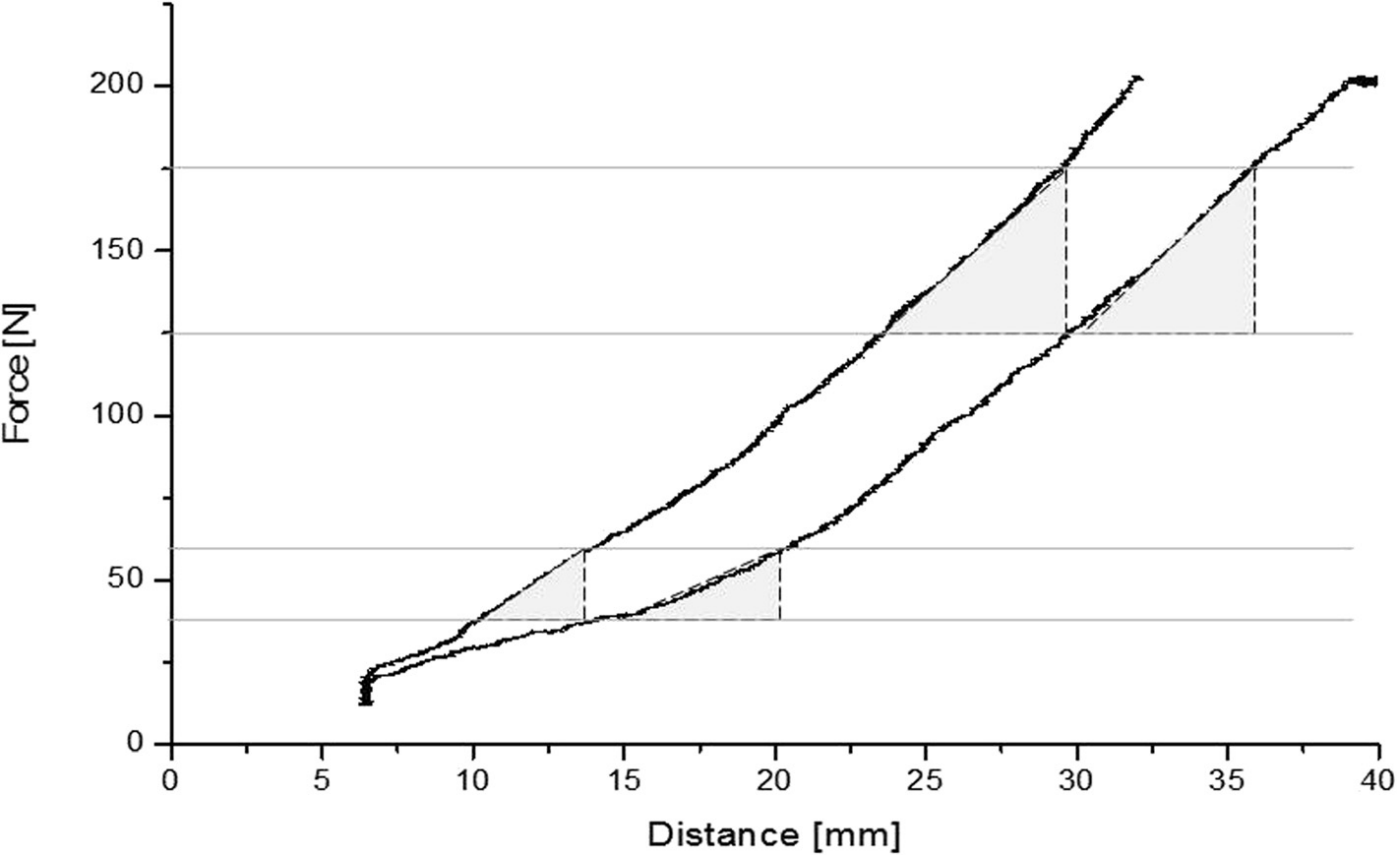
**Two typical load-deformation curves.** The left one is selected from a mechanically stable and the right one from a mechanically unstable ankle. The stiffness was calculated from the slopes (slope = ∆ Force [N]/∆ Distance [mm]) in the intervals from 40 to 60 N and 125 to 175 N. The stiffness is not different in the 125–175 N intervals but the stable subject’s 40–60 N slopes are steeper.

Finally, stress testing with the ankle arthrometer was conducted. Our ankle arthrometer is a non-radiographic device to objectively quantify ATD. It has already proven its validity in a cadaver experiment [30] and *in vivo* [28,32]. In principle, this apparatus pulls the heel anteriorly with respect to the fixed lower leg (8 mm/s, maximum force 200 N), and the respective distance is measured by a linear potentiometer (Additional file 1). From the resulting load-deformation curve, stiffness was calculated (Figure 3) [28,32,30]. The toe region (40–60 N) represents the tibiotalar translation (ATD) while the stiffness in the upper region (125–175 N) of the load-deformation curve indicates the rigidity of the ankle and its encompassing soft tissues with the talus already anteriorly translated to its end position [28,32]. Ankle arthrometer calculations were based on the mean values obtained from three consecutive measurements in each ankle (three trial average). Analyzes were based on one ankle per person. If both ankles were CAI, further analyses were focused to the side performing worse in the manually performed ATD test (11 subjects). In 15 subjects, there was no side difference and the side to be further considered was randomly chosen.

### Grouping

In line with our previous work [13], we diagnosed MAI when FAI symptoms were present and manual ankle stress testing revealed mechanical ankle instability (Figure 1). Respectively, 15 FAI subjects, 11 MAI subjects, and 15 MAI patients were grouped to test known group validity. Further analyses (diagnostic validity, correlation, and eta) were based on two groups (15 FAI vs. 26 MAI) combining the MAI subgroups (Figure 2).

### Statistical analysis

The SPSS statistical package 20.0 for windows (SPSS Inc., Chicago, IL) was used for statistical analysis. The Kolmogorov-Smirnov test was performed to test for normal distribution. Known group validity [30] was evaluated by comparing the arthrometer results between FAI (*n* = 15), MAI subjects (*n* = 11), and MAI patients (*n* = 15) using a one-way analysis of variance with Turkey’s post hoc procedure. Post hoc analyzed power for the number of subjects within the groups was 1-ß = 0.998 with α = 0.05. Eta for the relation between manual and arthrometer ATD testing was calculated. A receiver operating characteristic curve (ROC) analysis was calculated to determine which 40–60 N stiffness cut-off value was most suitable to discriminate between the mechanically stable (*n* = 15) and the mechanically unstable (*n* = 26) ankles. As a result of the ROC analysis, the diagnostic validity parameters of the arthrometer (sensitivity, specificity, positive, and negative predictive value) were calculated. The interaction of FAI (*n* = 15) and MAI (*n* = 26) was determined by a Pearson correlation analysis between the individual ankle arthrometer findings and the respective FAAM-G results. Tests were considered significant at a level of *p* < 0.05.

## Results

**Figure 4:**
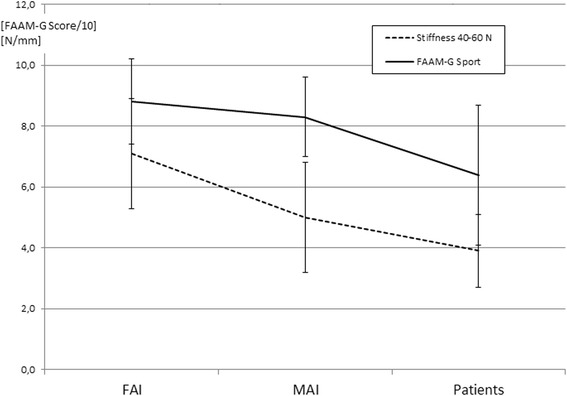
**Mean + SD demonstrating the FAAM-G scores/10 and the respective stiffness.** Mean + SD demonstrating the FAAM-G scores/10 and the respective stiffness in the low region (40–60 N) of the load-deformation curves obtained from the ankle arthrometer. The *p* values can be extracted from Table 3.

**Figure 5:**
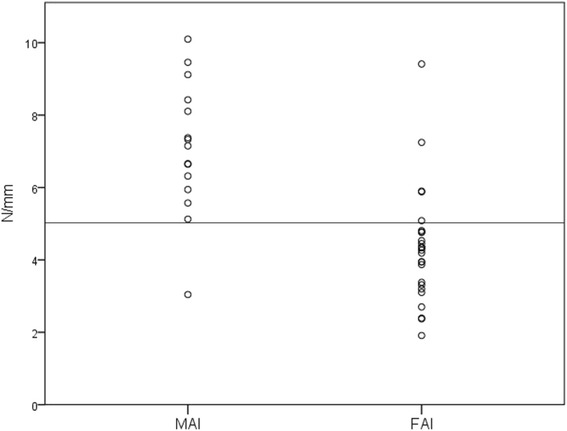
**Distribution of stiffness values.** Individual 40–60 N stiffness values for the functional ankle instability (FAI) and the mechanical ankle instability (MAI) group. The line (5.1 N/mm) represents the best cut off value (error classification rate = 11%) to discriminate between FAI and MAI with a sensitivity of 81% and a specificity of 93%.

The mean values of the 40–60 N stiffness analyses and for both FAAM-G subscales were highest in the FAI group and lowest in the patients (Tables 2 and 3, Figures 4 and 5).

**Table 2:** **Stiffness and FAAM-G results when categorized by manual instability testing; values are given as [N/mm]**

**Classification**	**No.**	**Stiffness 40–60 N**	**Stiffness 125–175 N**	**FAAM-G ADL**	**FAAM-G Sports**
		**(N/mm)**	**(N/mm)**		
FAI subjects	15	7.1 ± 1.8	9.4 ± 1.4	96.9 ± 5.6	88.2 ± 13.8
		[3.0–10.1]	[7.2–12.7]	[80–100]	[63–100]
MAI subjects	11	5.0 ± 1.8	8.5 ± 1.2	94.1 ± 7.1	83.8 ± 13.1
		[3.2–9.4]	[6.9–10.5]	[80–100]	[59–100]
MAI patients	15	3.9 ± 1.2	8.9 ± 2.8	88.1 ± 9.7	64.4 ± 22.7
		[1.9–5.9]	[4.7–13.7]	[71–100]	[16–100]

Means ± standard deviations [ranges] are presented.

Post hoc analyzed power for the number of subjects within the groups = 1-ß = 0.998with α = 0.05.

**Table 3:** **Stiffness and FAAM-G results when categorized by manual instability testing; respective statistical analyzes**

**Classification**		**Stiffness 40–60 N**	**Stiffness 125–175 N**	**FAAM-G ADL**	**FAAM-G Sports**
		***p*** **value**	***p*** **value**	***p*** **value**	***p*** **value**
FAI subjects vs.	MAI subjects	*0.006*	0.468	0.615	0.797
	MAI patients	*<0.001*	0.773	*0.011*	*0.002*
MAI subjects vs.	MAI patients	0.224	0.844	0.142	*0.024*

Significant findings are italicized.

In the low region, (40–60 N) of the load-deformation curves MAI subjects’ and patients’ ankles had significantly lower stiffness than FAI ankles (*p* = 0.006 and <0.001). There was no difference discernible between MAI subjects and patients’ (*p* = 0.224; Figure 4; Tables 2 and 3). In the 125–175 N load region of the load-deformation curves, no difference existed, when the groups were compared (all *p* > 0.468).

Eta for the relation between manual and arthrometer ATD testing was 0.628.

Regarding the FAAM-G subscale for activities of daily living, the FAI group was different from the patients’ group (*p* = 0.011). The sport subscale revealed lower scores for the patients when compared with both FAI and MAI subjects (*p* = 0.002 and 0.024; Tables 2 and 3).

**Figure 6:**
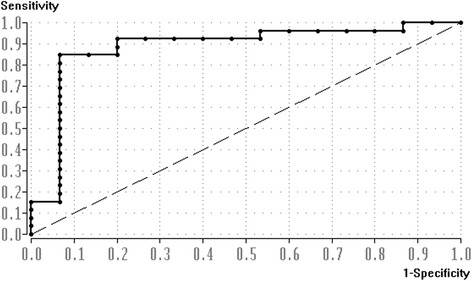
**Receiver operating characteristic (ROC) curve.** The true positive rate (sensitivity) is plotted against the false positive rate (1-specificity) for the different possible cut-off points.

ROC calculation (Figure 6) revealed the lowest error classification rate (11%) for a cut-off stiffness value of 5.1 N/mm. The resulting sensitivity (true positive rate) of the measurements was 81% and the specificity was 93%. The positive and negative predictive values were 96% and 94%, respectively.

The correlations between individual 40–60 N arthrometer stiffness values and FAAM-G scores for activities of daily living and sports were *r* = 0.286 (*p* = 0.07 and *r*=0.316 (*p*=0.04), respectively.

## Discussion

Known group validation confirmed that the assumed difference between mechanically stable (FAI) and unstable (MAI) subgroups of CAI can be measured by ankle arthrometry (*p* = 0.006 and <0.001). With the available numbers, patients and MAI subjects did not differ in this respect (*p* = 0.224). With a cut-off value of 5.1 N/mm a high diagnostic validity of the arthrometer is proven, supporting the results of a previous cadaver study [30].

CAI probably represents a continuum from mechanically stable to mechanically unstable ankles and dichotomous grading (FAI and MAI) is not representing the whole truth. However, experimental research found no evidence that MAI and FAI fall at different places within that continuum with MAI presenting more functional symptoms [13].

The FAAM-G results suggest that FAI subjects complain of less severe symptoms than MAI subjects and patients. However, only the sport subscale revealed relevant differences between MAI subjects and patients (*p* = 0.024). A poor to medium strength correlation was found between arthrometer and FAAM-G results. Our clinical [28] and recent experimental work [13] confirms these findings and demonstrates that both factors may “substantially interact” [9]. Previous research using ankle arthrometers and stress radiography also detected greater ATD in functionally unstable ankles [24,32].

A control group without previous ankle injury was not recruited for the present study. This comparison, however, was recently published demonstrating clear between group differences (*p* < 0.01) for the ankle arthrometry and FAAM-G [32]. An individual comparison to the uninjured side was not performed because current research has demonstrated evidence for a central dysfunction in CAI subjects affecting also the uninjured leg [36].

Even if there is still disagreement with its usefulness [19-23], manual stress testing is considered as standard to diagnose MAI [6,16-18,35,37]. Experimental cadaver studies found insufficient interrater reliability [18,23] but excellent intraobserver reliability (*r* = 0.94) for ATD and sensitivity and specificity, respectively, were 100% and 66.67% [18]. Validated against surgery and arthrography, the specificity and sensitivity of physical examination to detect an ankle ligament lesion were found to be 84% and 96%, respectively, and there was also a good interobserver agreement (kappa values 0.5 to 1.0) [16]. Intrarater reliability (*r* = 0.9; *p* < 0.001) for the manually performed ATD test is described to be excellent [38]. In an *in vivo* experimental study, the manually performed ATD demonstrated good sensitivity (74% or 83%, depending on the set standard) and strong correlation (rho = 0.62, *p* = 0.02) when compared with ultrasound laxity measurement [19]. Concluding from these results, we feel that the ATD test, specifically when performed by a single and experienced observer, is currently the most objective tool to differentiate mechanically stable from unstable ankles. Therefore, we selected this test as standard for diagnostic validity testing.

Resulting from the eta test, a “moderate to large” relation was proven between manually and arthrometer ATD testing. This indicates that the arthrometer is suitable for quantifying ATD instability. In general, it would be preferable to validate the arthrometer with another continuous measure, e.g., stress radiographs. However, the validity of stress radiographs *in vivo* is still under intensive debate [26,27] and was not performed due to ethical considerations (radiation). Additionally, an arthrometer validation against stress radiographs was already successfully performed previously in a cadaver experiment [30].

The major clinical implication of this study is the generation of a cut-off value to differentiate mechanical stable from mechanically unstable ankles in CAI. Only a weak relation between mechanical (arthrometer) and functional (FAAM-G) measures was demonstrated with more subjective limitations in MAI. Therefore, manual testing or arthrometry should be performed in all persons presenting with FAI symptoms because MAI persons seem to be “more prone to recurrent ankle sprains” [13] and need further mechanical support (tape, bandage, brace, or surgery).

### Limitations of this study

This study was planned and performed before the “International Ankle Consortium” published its “selection criteria for patients with chronic ankle instability in controlled research” [3]. However, our inclusion criteria are consistent with these criteria: (a) previous significant ankle sprain, (b) giving way, and/or recurrent sprain, and/or feelings of instability, and (c) a “self-reported foot and ankle function questionnaire is recommended”. With respect to the FAAM-G activities of daily living and sport subscales, cut-off values of 90% and 80%, respectively, are proposed as upper limits for inclusion [3]. Our FAI and MAI subject groups scored close to while the patients scored clearly below these limits. Our selection criteria excluded copers (no residual symptoms after an ankle sprain more than 1 year ago).

All participants in our study were male, and the results may not be generalizable to females. But this is not a limitation but rather strength. Relevant differences between genders were reported using another ankle arthrometer [29]. Therefore, including females could likely bias the results and remains the scope for further research.

## Conclusions

Compared with manual ankle instability testing, our ankle arthrometer proved to be a valid instrument to differentiate mechanically stable from mechanically unstable ankles in male subjects and patients. There are several benefits of the ankle arthrometer stiffness assessment to recommend its use in clinical practice. The ankle arthrometer provides rater independent, continuous data and is radiation free. The ankle arthrometer better addresses the mechanical component, whereas the FAAM-G better addresses the functional component. We could demonstrate only a weak interaction (correlation) between functional (FAAM-G) and mechanical (arthrometry) measures. For further clinical practice and scientific CAI research, both measures should be collected.

## Additional file

Additional file 1:
**Video clip to demonstrate the make, model, and function of the used ankle arthrometer.** Here, an ankle model is placed in the arthrometer for better demonstration of the principle of its functioning.

## Abbreviations

FAAM-G: Foot and ankle ability measure-German version
CAI: Chronic ankle instability
FAI: Functional ankle instability
MAI: Mechanical ankle instability
ATD: Anterior talar drawer
MRI: Magnetic resonance imaging
BMI: Body mass index
ROC: Receiver operating characteristic
